# Pruning or tuning? Maturational profiles of face specialization during typical development

**DOI:** 10.1002/brb3.464

**Published:** 2016-04-15

**Authors:** Xun Zhu, Ramesh S. Bhatt, Jane E. Joseph

**Affiliations:** ^1^ Department of PsychologyShihezi UniversityXinjiangChina; ^2^ Department of NeurosciencesMedical University of South CarolinaCharlestonSouth Carolina29425; ^3^ Department of PsychologyCollege of Arts and SciencesUniversity of KentuckyLexingtonKentucky40506

**Keywords:** Development, face perception, fMRI

## Abstract

**Introduction:**

Face processing undergoes significant developmental change with age. Two kinds of developmental changes in face specialization were examined in this study: specialized maturation, or the continued tuning of a region to faces but little change in the tuning to other categories; and competitive interactions, or the continued tuning to faces accompanied by decreased tuning to nonfaces (i.e., pruning).

**Methods:**

Using fMRI, in regions where adults showed a face preference, a face‐ and object‐specialization index were computed for younger children (5–8 years), older children (9–12 years) and adults (18–45 years). The specialization index was scaled to each subject's maximum activation magnitude in each region to control for overall age differences in the activation level.

**Results:**

Although no regions showed significant face specialization in the younger age group, regions strongly associated with social cognition (e.g., right posterior superior temporal sulcus, right inferior orbital cortex) showed specialized maturation, in which tuning to faces increased with age but there was no pruning of nonface responses. Conversely, regions that are associated with more basic perceptual processing or motor mirroring (right middle temporal cortex, right inferior occipital cortex, right inferior frontal opercular cortex) showed competitive interactions in which tuning to faces was accompanied by pruning of object responses with age.

**Conclusions:**

The overall findings suggest that cortical maturation for face processing is regional‐specific and involves both increased tuning to faces and diminished response to nonfaces. Regions that show competitive interactions likely support a more generalized function that is co‐opted for face processing with development, whereas regions that show specialized maturation increase their tuning to faces, potentially in an activity‐dependent, experience‐driven manner.

## Introduction

**Figure 1:**
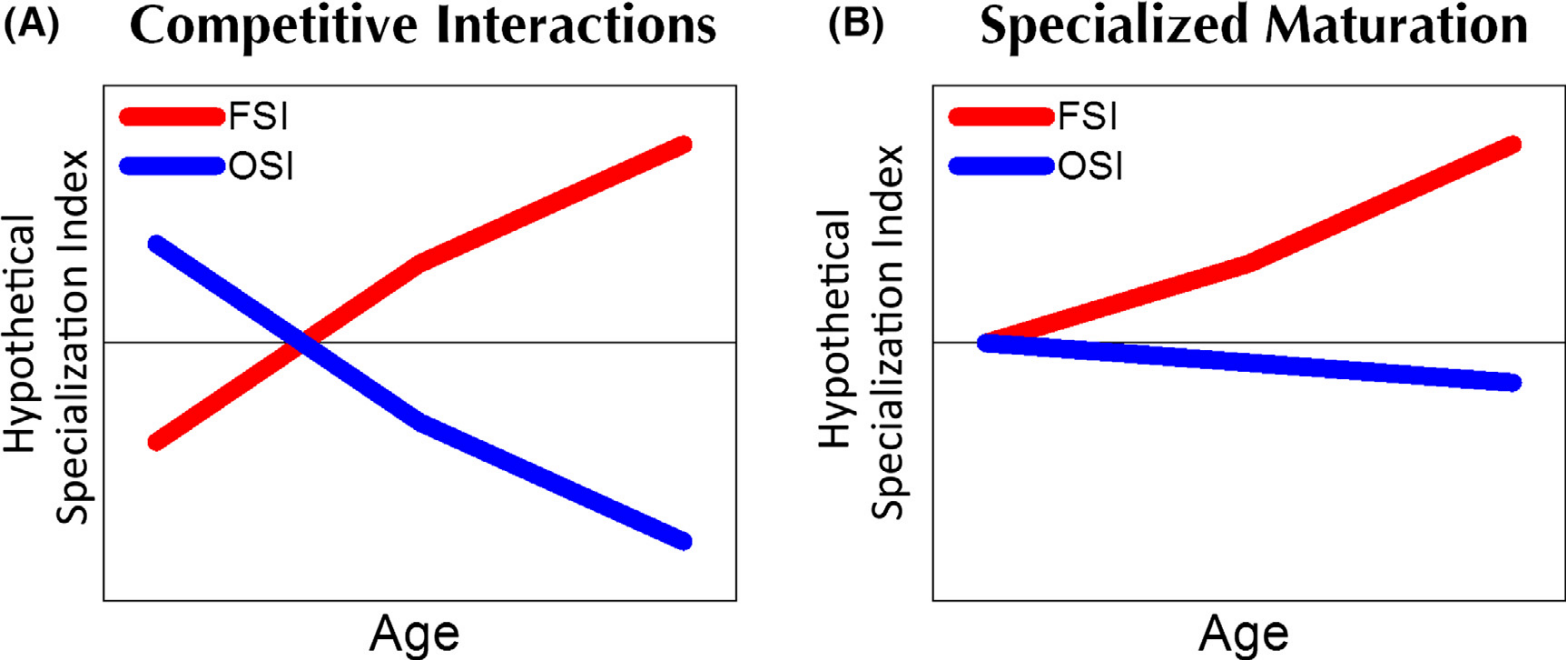
Hypotheses associated with different accounts of face and object processing development. Hypothetical specialization indices are shown on the *y*‐axis and age is shown on the *x*‐axis. A face specialization index is shown in red; an object specialization index is shown in blue. Competitive interactions are characterized by increased specialization for faces with age but decreased specialization for objects with age in the same brain region. Specialized maturation is characterized by increased specialization for faces with age but no developmental change for objects in the same brain region.

The development of functional brain architecture that supports specialized cognitive functions, like face processing, is continually debated. In the IS (Interaction Specialization) account of functional brain development (Johnson 2005), functional specialization is achieved by a dynamic interplay of changes in brain‐to‐function mappings. One key feature of the IS account is the presence of competitive interactions in these mappings such that a given brain region increases its tuning to a particular category while pruning back responses to other categories (Fig. 1A). A consequence of this is that a particular brain region may show a preference for nonface categories earlier in development, or show no specific domain preference, but as the brain matures, the region becomes more narrowly tuned to faces and the preference for nonfaces will diminish with development.

Testing the developmental time course of both faces and nonfaces is critical in order to distinguish among different constraints on the development of functional specialization. As shown in Figure 1B, an alternative to competitive interactions is specialized maturation of a brain region, or increased tuning to faces but no pruning of responses to non‐preferred categories. This outcome would be predicted by the Maturational account (discussed in Johnson 2005; Joseph et al. 2011) or Constructivist viewpoints (Quartz 1999). In these alternative accounts, the primary process is increased tuning of a region to faces but minimal pruning back of nonpreferred responses. The maturational viewpoint posits that this increased tuning is determined genetically whereas the constructivist view posits that increased specialization is accomplished through Hebbian learning and dendritic growth, but not synaptic loss. Although Johnson (Johnson 2005, 2011) has suggested that there is little neurobiological evidence for the constructivist account, Farah et al. (2000) provided evidence for a strong form of the maturational viewpoint in their study of a boy who acquired a lesion at 1 day of age and showed the classic neurobehavioral profile of prosopagnosia when tested at age 16: deficits in face but not object processing and damage to bilateral occipital and occipito‐temporal cortex. They concluded that “prior to visual experience, we are destined to carry out face and object recognition with different neural substrates. This in turn implies that some distinction between face and object recognition, and the anatomical localisation of face recognition, are explicitly specified in the genome” (p. 122) and “the distinction between faces and other objects, and the localisation of faces relative to other objects, is fully determined prior to any postnatal experience” (p. 117). However, if the maturational account is viable then children should show the same preference for faces in occipito‐temporal cortex (or in the more specific “fusiform face area,” FFA; (Kanwisher et al. 1997) as adults do. Moreover, if portions of the fusiform gyrus are completely devoted to face processing from birth, then other objects should not recruit that region. However, neither of these conditions holds, because the FFA responds to objects other than faces (Joseph and Gathers 2002) and perceptual expertise with other categories can recruit the FFA (for example “Greebles” (Gauthier et al. 1999); chess configurations (Bilalic et al. 2011)). Moreover, as discussed more below, specialization for faces increases with age. This suggests that a strong form of the maturational viewpoint may not be viable. We instead investigate the constructivist idea that a brain region may increase in tuning to faces without pruning of nonface responses.

Systems‐level approaches to cortical development that use fMRI or functional connectivity analyses have not yet distinguished between the IS and alternative accounts. In an in‐depth treatment of this topic, Joseph et al. (2011) outline the conditions that should be met in order to support the IS versus maturational/constructivist accounts. Nearly all of the fMRI studies that have examined developmental changes in functional organization for basic face processing (i.e., not including higher social cognitive functions such as facial emotion, social evaluation, mentalizing; (Aylward et al. 2005; Gathers et al. 2004; Golarai et al. 2007, 2010; Haist et al. 2013; Joseph et al. 2011; Passarotti et al. 2003; Peelen et al. 2009; Pelphrey et al. 2009; Scherf et al. 2007, 2011) have supported a pattern of increased specialization for faces with age, indicated by increased magnitude or extent of FFA response to faces versus nonfaces. However, this outcome would be predicted by both the IS and alternative viewpoints, as shown in Figure 1. For both competitive interactions and specialized maturation, the relative difference between face and nonface response increases with age, but the difference between the two accounts is driven by the response to nonfaces as a function of age, *in the same brain region where face specialization increases with age*. Specialized maturation predicts no change in nonface response with age whereas competitive interactions predict a decrease in nonface response with age. Prior studies have either examined the relative response to faces versus nonfaces (which is inconclusive with respect to the IS or maturational accounts) or showed no developmental change for nonfaces in regions that are object‐preferential or not preferential for faces (Golarai et al. 2007, 2010; Scherf et al. 2007, 2011; Peelen et a0l. 2009; Joseph et al. 2011). Also, these studies did not examine age trends separately for faces and nonfaces *in the same brain region* which is the only way to distinguish these two accounts. This study accomplishes this, which represents a significant advance in teasing apart the constraints on cortical development of face expertise. In the present conceptualization, the strongest evidence for competitive interactions would be that the *same* brain region demonstrates a developmental tradeoff in tuning for different categories (Fig. 1).

One study has provided some evidence for the phenomenon of tradeoffs in tuning for different categories. Cantlon et al. (2011) showed a face‐specific response in the right FFA in 4 to 5 year olds, suggesting very early specialization of this cortical region for faces. Interestingly, though, face matching accuracy (measured outside of the scanner) was negatively correlated with the right FFA's response to letters, but was not correlated with the response to faces. In other words, face‐matching performance at this very early age was more strongly linked to changes in the FFA's tuning to the *nonpreferred category* (letters) than tuning to the preferred category (faces). The authors interpreted this result as driven by pruning back responses to the nonpreferred category with development, rather than driven only by increased tuning to the preferred category.

Because so few studies have investigated developmental changes in both pruning and tuning processes at a systems level with fMRI, this study will examine both phenomena. It is possible that some regions are indeed largely destined to process faces without competition from other nonface categories, whereas other regions co‐opt the kind of processing that is initially applied to objects, or to both faces and objects more generally, in order to fine tune face processing. To our knowledge, no fMRI studies have examined this possibility.

To delineate among different accounts of development of face specialization, this study examines face and object specialization changes during childhood (younger group: 5–9 years; older group: 9–12 years) and in adulthood in a network of regions implicated in face processing in adults. In order to examine age trends for faces and nonfaces (objects) simultaneously, the present study compares each category to an active control condition, viewing visual textures, which enables scaling each category's response to a common activation baseline rather than comparing faces and objects directly. This allows us to distinguish between competitive interactions and specialization maturation profiles. The study also explores various approaches to index face and object specialization. The measure of face (or object) specialization used in this study scales the differential response to faces versus nonfaces to the maximum value for each subject in each region, thereby controlling for age‐related magnitude differences when measuring face or object selectivity. To our knowledge, other studies did not scale responses in this manner. Because the goal is to examine how face specialization develops, the face‐preferential ROIs (regions of interests) are defined in adults, as an estimate of the endpoint of the developmental process. Adults are expected to demonstrate more face specialization than children given prior literature findings. ROIs are defined using a subset of the adults, but hypotheses about IS and the alternative accounts are tested in the full sample of adults and children to maximize statistical power. Because the full sample was not used to define the ROIs, hypothesis testing was separate from ROI definition. However, we also conduct analyses using the smaller sample of adults and all children in order to test hypotheses completely independently from ROI definition (Kriegeskorte et al. 2009).

The analytic approach examines age group effects in the adult face network (using ANOVAs conducted in ROIs) to explore interactions of age and category specialization (face or object specialization index) in face preferential regions. Although the ROI are defined in adults, it is possible that children recruit face‐ regions that are different from the regions recruited by adults, as demonstrated in a prior study (Joseph et al. 2011). The finding that children recruit different regions from adults is consistent with the IS account in that some regions lose functionality over development to make way for different regions to be specialized. This study does not test this particular hypothesis directly, but will explore additional regions recruited for faces in children, both at the group level and at the individual‐subject level.

The IS account will be supported by findings of competitive interactions (Fig. 1A) in regions that are specialized for faces in adults; that is, there will be decreased object‐specialization (pruning) and increased face‐specialization (tuning) with age in the same brain region. Although Figure 1A illustrates greater object‐ than face‐specialization in younger children, this is not necessary to support competitive interactions. However, it would be compelling to show that a brain region is initially recruited for objects, but then becomes tuned to faces with development. The Maturational and Constructivist viewpoints will be supported by findings of specialized maturation (Fig. 1B); that is, there will be increased face‐specialization (tuning) but no changes in object specialization with age (no pruning). This study will reveal whether pruning or tuning mechanisms are more prominent in the development of specialized brain networks for face processing. Knowing which regions show a specialized maturation profile versus competitive interactions has strong implications for the understanding the degree of neuroplasticity present in the brain for face processing. In turn, this knowledge could inform behavioral interventions for individuals who encounter difficulty with various face capacities, as in Autism Spectrum Disorder, Williams Syndrome or developmental prosopagnosia by targeting the neurobehavioral domains that are most likely modifiable through learning and experience.

## Method

### Participants

Forty‐eight healthy right‐handed children (23 males, 5.5–12 years, mean age = 8.7 years, SD = 1.94) were enrolled in and completed the study, but due to excessive head motion (i.e., more than 20% time points with relative displacement >0.5 mm), data from eight participants were eliminated. The remaining 40 child data sets were separated into two age groups similar to the age groups used in prior studies (Gathers et al. 2004; Joseph et al. 2011, 2012): 21 younger children (seven males, 5.5–8.4 years, mean = 7.1 years, SD = 0.86) and 19 older children (11 males, 9.3–11.7 years, mean = 10.5 years, SD = 0.59). This age grouping is also well motivated based on the finding that some aspects of face processing show significant developmental changes around age 10 or are already adult‐like by this age (Diamond and Carey 1977; McKone et al. 2012). Twenty three of these subjects’ data were used as healthy controls reported in (Joseph et al. 2015b), but that paper did not analyze face‐ and object‐specialization indices as a function of different kinds of maturational profiles (specialized maturation or competitive interactions) as in this study.

Fifty‐nine healthy right‐handed adult volunteers (29 males; mean age = 26.5 years, SD = 6.0, range 18–42) were compensated or received course credit for participation. Due to excessive head motion (max absolute motion >1.75 mm, or half the voxel size), data from eight participants were eliminated, leaving 51 adult participants (26 males; mean age = 26.7 years, SD = 6.1, range 18–42). Results from the adult group have been reported in (Collins et al. 2012), but the analysis of face‐ and object‐specialization indices in this study was not reported in that prior study.

No participants reported neurological or psychiatric diagnoses or pregnancy and all provided informed consent before participating. All procedures were approved by local Institutional Review Board.

### Stimuli and procedure

Three different categories of visual stimuli were used in the present face localizer task: face photos, manmade object photos, and texture patterns. These visual stimuli were organized into a block‐design task which consisted of nine 17.5 sec blocks (three for face, three for object, and three for texture) with 12.5 sec fixation period interleaved. During each task block, 10 different yearbook face photos, manmade objects, or texture pattern were shown. Each photograph was presented for 1000 msec following a fixation of 750 msec. During each fixation block participants saw a black crosshair on a white background. Participants were asked to press a button each time a stimulus appeared using a fiber‐optic response pad (MRA Inc., Washington, PA) to ensure attentive processing. All groups showed a high rate of response (Adults: 97.9%; older children: 91.5%; younger children: 84.8% (due to a technical issue three adults’ and one younger child's responses were not recorded and not included in above accuracy calculation), indicating that even the younger children attended to the stimuli. Each participant completed one face localizer run and four other functional runs of a matching task in counterbalanced order. Results from the matching task are not reported here.

### fMRI Data acquisition and analysis

Images were acquired on a Siemens 3T Trio MRI system (Erlangen, Germany) at two different sites, but the hardware and software versions were identical across sites. Scanning included a 109‐volume (272.5 sec) whole‐brain functional scan (gradient echo EPI; TE = 30 msec, TR = 2500 msec, flip angle = 80°, FOV = 22.4 cm × 22.4 cm, interleaved acquisition of 38 axial contiguous 3.5‐mm slices) and a T1‐weighted anatomical scan (MPRAGE; TE = 2.56 msec, TR = 1690 msec, TI = 1100 msec, FOV = 25.6 cm × 22.4 cm, flip angle = 12°, 176 contiguous sagittal 1‐mm thick slices). Field map information (to correct geometric distortions caused by static‐field inhomogeneity) was also collected. E‐prime software (version 1, www.pstnet.com; Psychology Software Tools) running on a Windows computer connected to the MR scanner presented visual stimuli and recorded the time of each MR pulse, visual stimulus onset, and behavioral responses.

Preprocessing and statistical analysis were conducted using FSL (v. 4.1.7, FMRIB, Oxford University, Oxford, U.K.). For each subject, preprocessing included geometric distortion correction, motion correction with MCFLIRT, spatial smoothing with a 7‐mm FWHM Gaussian kernel and temporal high‐pass filtering (cutoff = 100 sec). Statistical analyses were then performed at the single‐subject level (FEAT v. 5.98). Each scan was modeled with three EVs (explanatory variables; faces, objects, and textures) convolved with a double gamma HRF, and a temporal derivative. Baseline blocks were not explicitly modeled. For the analysis of children's data, in addition to including six head motion parameters (three translational, three rotational) as confound EVs, we further included a spike EV which reflected all time points with relative displacement >0.5 mm to regress out the motion artifacts. We opted to use a single spike EV rather than scrubbing (multiple spike EVs), because the number of time points we had to detect any single effect (e.g., Face, Object or Texture activation) is relatively smaller than the number of time points used in other designs (e.g. continuous resting state) when scrubbing is typically applied. Since scrubbing basically removes the time point in question, this approach could seriously degrade power to detect the effects of interest in this study.

### Regions‐of‐interest definition

Half of the adult subjects (*n* = 25) were randomly selected (13 males, 20–41 years of age) to define the ROIs for the present study. Face > object, face > texture, and face > fixation statistical maps were calculated at the individual subject level, then a mixed‐effects group analysis (using FLAME 1 + 2) yielded group‐level statistical parametric maps for face > fixation and face > object contrasts in the 25 adults. For each adult subject, contrast maps were registered via the subject's high‐resolution T1‐weighted anatomical image to the adult MNI‐152 template (12‐parameter affine transformation; FLIRT) yielding images with spatial resolution of 2 mm^3^. Group contrast images were thresholded using clusters determined by *Z* > 2.3 and a corrected cluster significance threshold of *P* = 0.05. Face‐preferential regions were defined by the logical combination (Joseph et al. 2002) of face > object and face > texture contrasts, with cluster local maxima based on the face > object contrast. We identified 14 local maxima across the brain in this step, and the ROIs were defined as 7 mm‐radius spheres centered on these local maxima. A 7 mm radius sphere was chosen because our image smoothing kernel was 7 mm.

### Regions of interest analysis

For each ROI defined in the 25 adults, % signal change relative to fixation was extracted for each event type (faces, objects, textures) from the first level analysis (using FSL's Featquery tool) for each subject (51 adults, 19 older children, 21 younger children). Percent signal change for the three categories (face, object, texture) for each subject and region was then used to compute an FSI (face specialization index) and an OSI (object specialization index). As there is no standard approach to computing a specialization index, we explored different formulas (outlined in Appendix S1). Based on its distributional properties and better face validity, we adopted FSI_B_ and OSI_B_ for the primary analyses:

$${\text{FSI}}_{B}=\frac{{\text{Face}}_{adj}-\left({\text{Object}}_{adj}+{\text{Texture}}_{adj}\right)/2}{\text{Max}({\text{Face}}_{adj},{\text{Object}}_{adj},{\text{Texture}}_{adj})}$$

where

$${\text{Face}}_{adj}=F_{pc}+\left|\text{minimum}\left(F_{pc},O_{pc},T_{pc}\right)\right|;$$

$${\text{Object}}_{adj}=O_{pc}+\left|\text{minimum}\left(F_{pc},O_{pc},T_{pc}\right)\right|;$$

$${\text{Texture}}_{adj}=T_{pc}+\left|\text{minimum}\left(F_{pc},O_{pc},T_{pc}\right)\right|;$$

and *F*_*pc*_, *O*_*pc*_, *T*_*pc*_ are percent signal change for faces, objects, or textures, respectively, relative to baseline.

Similarly,

$${\text{OSI}}_{B}=\frac{{\text{Object}}_{adj}-\left({\mathrm{Face}}_{\mathrm{adj}}+{\text{Texture}}_{\mathrm{adj}}\right)/2}{\text{Max}({\text{Face}}_{\mathrm{adj}},{\text{Object}}_{\mathrm{adj}},{\text{Texture}}_{\mathrm{adj}})}$$

This formula is a modification of that used by Joseph et al. (2011) with an adjustment for negative values described by Simmons et al. (2007). This formula scales the face‐ (or object‐) preferential response to the maximum value of Fpc, Opc, and Tpc which addresses potential age differences in BOLD signal magnitude. FSI_B_ and OSI_B_ will range from −1 to 1, with more positive values indicating greater specialization for faces (or for objects in the case of OSI_B_) and more negative values indicating a preference for the other two categories.

Although the specialization index is the primary‐dependent variable in this study, we also determined whether percent signal change relative to baseline for the face condition was different from 0 in each age group separately for each ROI. This analysis is important for illustrating that even if the FSI is 0 for an age group, this does not imply that there was no activation in a region. A specialization index of 0 indicates that there was no preferential activation for faces (or objects), but the percent signal change could be greater than 0. To test this, a one‐sample *t*‐test was used to determine whether percent signal change was greater than 0 for a given region and age group.

### Analysis of FFA size

Given that other studies have reported developmental changes in FFA extent (Golarai et al. 2007, 2010; Scherf et al. 2007; Peelen and Kastner 2009; Haist et al. 2013), the present study explored whether FSI_B_ or OSI_B_ would be different for different FFA sizes. A series of right FFAs that differed in size were generated from the Face > Object contrast in the 25 adults that were used to define ROIs for the primary analysis by applying different statistical thresholds (from *z* = 1.4 to *z* = 3.0 step = 0.1, uncorrected, no cluster correction). The reason for using *z* = 1.4 as the minimum threshold was that this was the lowest threshold at which a succinct region consistent with the FFA emerged. At lower thresholds the FFA was connected with activations around lateral occipital cortex. BOLD signals were extracted and FSI_B_ and OSI_B_ were further examined as a function of FFA size using univariate ANOVA to test if the main effect of age was significant for each FFA size and one‐sample *t*‐tests to determine whether FSI_B_ was different from 0 for each FFA size and age group.

### Analysis of individual‐subject face‐preferential responses

One concern with using a group‐defined ROI to assess degree of face specialization is that the BOLD signal is averaged and smoothed, thereby potentially diluting face specialized responses in some individuals. This may especially be a concern with developmental studies given that some studies report different loci of activation to faces in children compared to adults (Gathers et al. 2004; Joseph et al. 2011; Passarotti et al. 2003). To address this, we isolated face‐preferential voxels for each individual subject. Face‐preferential voxels were defined from the Face > Object contrast in each subject within an anatomically defined right fusiform ROI (from the AAL atlas (Tzourio‐Mazoyer et al. 2002)). Individual right fusiform masks were generated in subject's native EPI space based on the inverse transformation matrix used to register native space to MNI atlas space (which was generated in the previous registration step). Individual‐subject voxels that survived an uncorrected threshold of *z* = 3.1, or *P* < 0.001 (similar to the approach used by Golarai et al. (2007)) were then submitted to ANOVAs to determine age effects on the number of significant voxels, location of the peak voxel, and degree of face and object specialization among surviving voxels.

## Results

### Group‐level activation results

**Figure 2:**
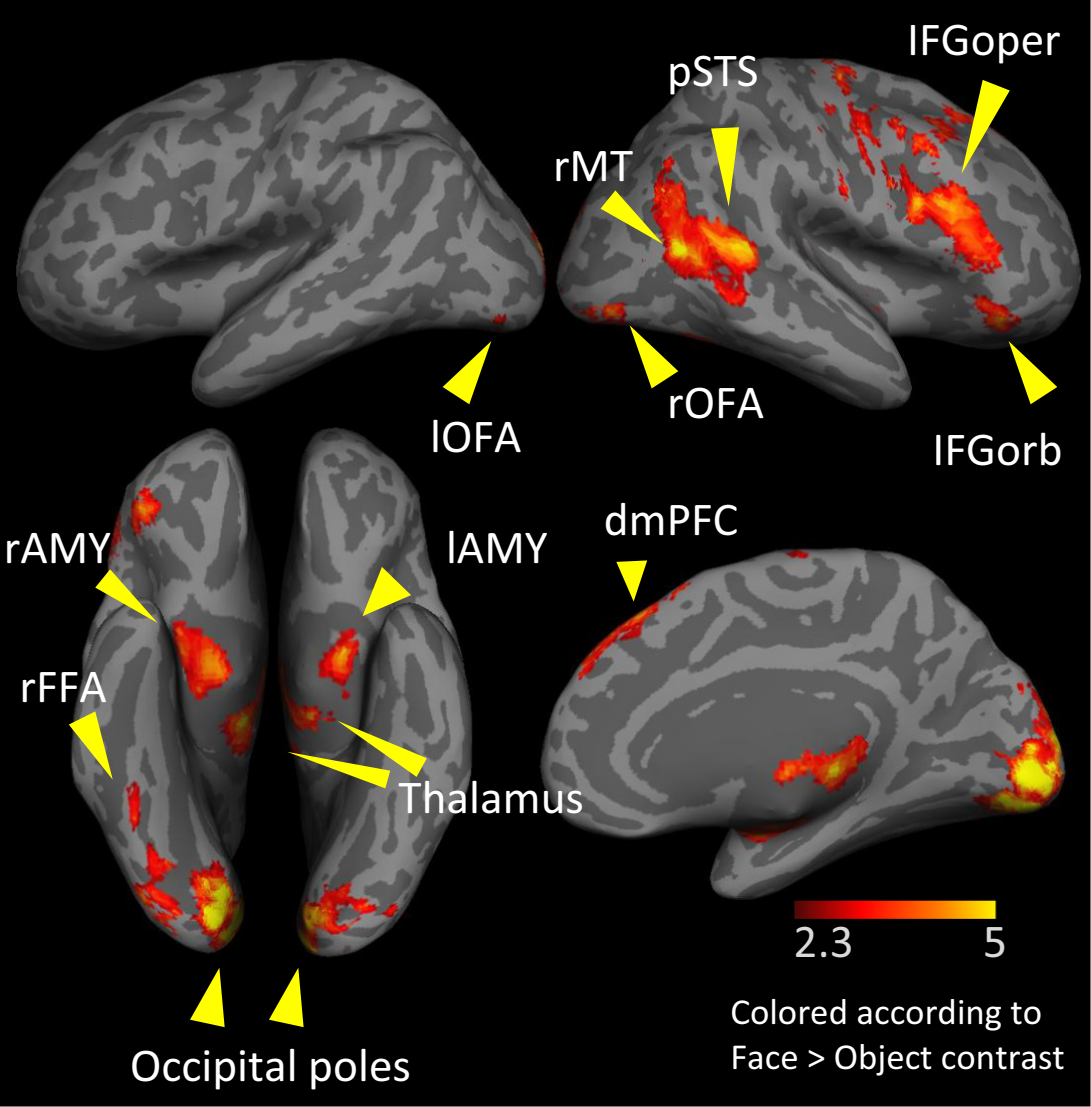
ROIs (regions of interest) used in the present study. ROIs were defined as face‐preferential in half of the adult sample, using GRF cluster correction, *P* < 0.05 (see text and Table 1 for more details). l, left; r, right; AMY, amygdala; FFA, fusiform face area; IFG‐oper, inferior frontal gyrus, pars opercularis; IFG‐orb, inferior frontal gyrus, pars orbitalis; OFA, occipital face area; pSTS, posterior superior temporal sulcus; MT, middle temporal gyrus; dmPFC, dorsomedial prefrontal cortex.

Face‐ preferential regions for the 25 adults used for ROI definition are outlined in Table 1 and illustrated in Figure 2. As expected, face‐preferential regions included the right FFA and OFA (occipital face area), right IFG (inferior frontal gyrus), dmPFC (dorsomedial prefrontal cortex), right pSTS (posterior superior temporal gyrus), right posterior MT (middle temporal cortex), bilateral AMG (amygdala), and bilateral occipital pole, as well as other brain regions. These regions served as ROIs in which FSI_B_ and OSI_B_ were further examined.

**Table 1:** Regions of interest (listed from anterior to posterior) isolated from a subset of the adults and results of the ROI analyses

Region	MNI coordinate (in mm, max *z* of Face > Obj)			Main effect of		Category × age interaction	Simple effect of age on	
				Category	Age		FSI	OSI
	*x*	*y*	*z*	*F*(1, 88) =	*F*(2, 88) =	*F*(2, 88) =	*F*(1, 88) =	*F*(1, 88) =
IFG_orb_	46	32	−14	ns	0.09^a^	0.028	0.001	ns
dmPFC	8	32	56	0.07^a^	ns	0.04	0.089^a^	ns
IFG_oper_	44	12	30	0.03	ns	0.01	0.023	0.042
rAMY	20	−10	−12	0.001	ns	0.032	0.03	ns
lAMY	−20	−14	−16	ns	ns	0.002	0.003	0.018
rTha	24	−24	2	ns	ns	0.052^a^	0.043	ns
lTha	−10	−26	0	ns	ns	0.059^a^	0.034	ns
pSTS	48	−44	10	<0.001	ns	0.048	0.008	ns
FFA	44	−54	−22	0.017	ns	0.093^a^	0.019	ns
MT	58	−60	14	<0.001	ns	0.047	0.093^a^	0.056^a^
rOFA	34	−78	−16	ns	ns	0.014	0.026	0.086^a^
lOFA	−26	−84	−22	ns	ns	ns	ns	ns
rOP	10	−88	−4	<0.001	ns	ns	ns	ns
lOP	−6	−100	4	<0.001	ns	ns	ns	ns

AMY, amygdala; dmPFC, dorsomedial prefrontal cortex; FFA, fusiform face area; IFGoper, inferior frontal gyrus, pars opercularis; IFGorb, inferior frontal gyrus, pars orbitalis; l, left; MT, middle temporal cortex; OFA, occipital face area; OP, occipital pole; pSTS, posterior superior temporal sulcus; r, right; Tha, Thalamus.

a Marginally significant effect (0.05 < *P* < 0.10); ns, not significant.

**Figure 3:**
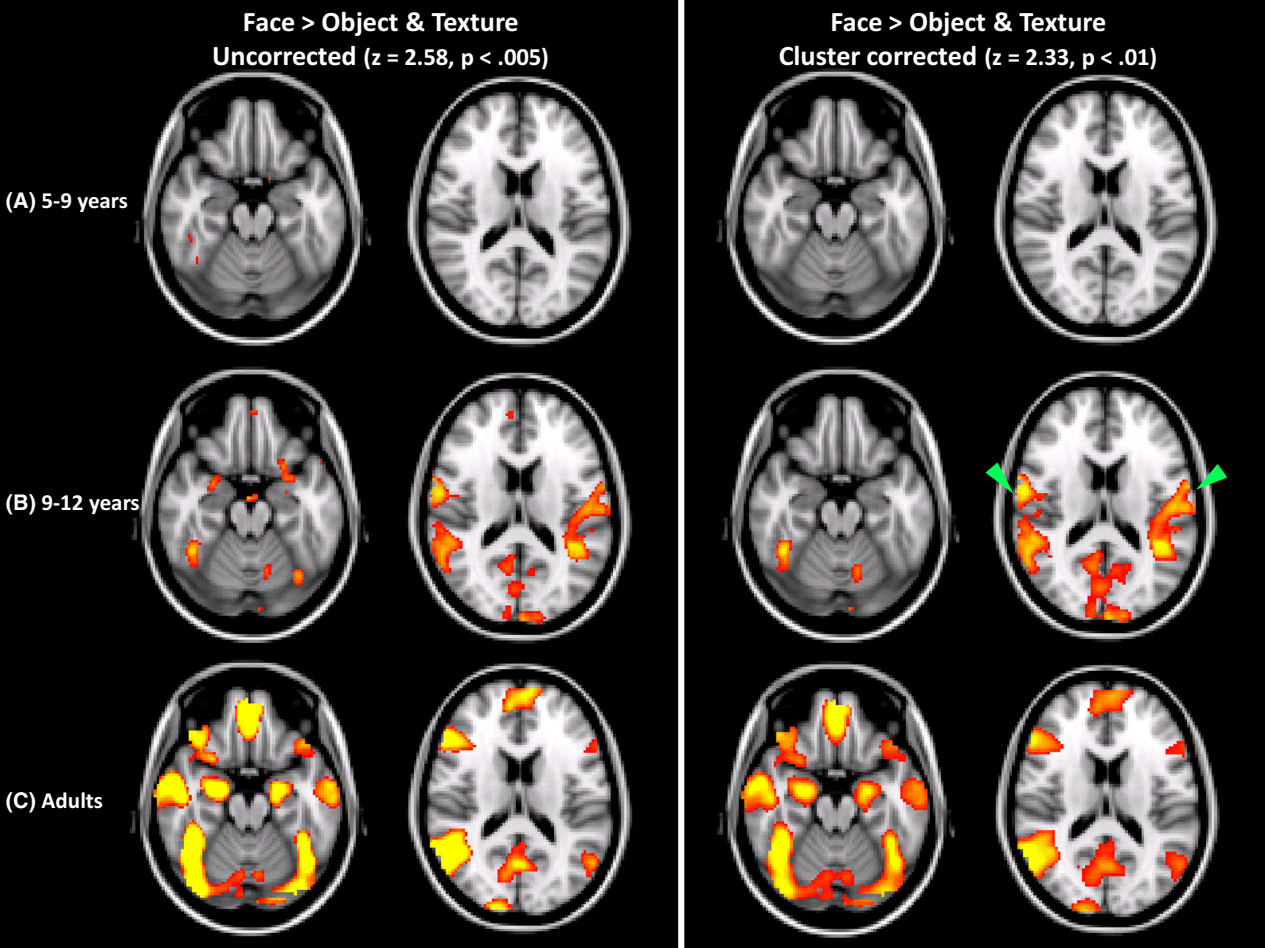
Face versus Object and Texture activation for each of three age groups: (A) younger children, (5–9 years), (B), older children, (9–12 years), (C) adults. The left panel shows results for an uncorrected threshold and the right panel shows results using cluster correction. The green arrows indicate regions of activation in older children that were unique to that age group.

We also examined the Face versus Object and Texture activation map (which is similar to face‐preferential activation) in each age group separately in order to examine whether younger children recruit different regions than older children or adults as reported by Joseph et al. (2011). Figure 3 shows the statistical parametric map from the contrast Face versus Object and Texture in each age group separately (all adults were included here). The left side of the figure shows activation that survived an uncorrected threshold and the right side shows activation that survived cluster correction. One obvious point from these results is that younger children show no activation at corrected thresholds, but show some, albeit scant, activation at an uncorrected threshold, including the right FFA. Most of the activations in older children overlapped with activations in adults with the exception of fairly extensive bilateral operculum activations, near primary auditory cortex (Fig. 3B, green arrows). Notably, though, much of the activation in adults is missing in both older and younger children including the extensive occipital, anterior temporal, frontal and AMG activation.

### Regions of interest results

In each ROI, a 2 (Category: FSI_B_, OSI_B_) × 3 (Age: younger children, older children, adults) mixed ANOVA was conducted (with age as a between‐subjects variable and category as a repeated measure) to determine whether face or object specialization varied across age. Each maturational profile predicts that there will be a Category × Age interaction, but the presence of an interaction by itself would not distinguish between competitive interactions and specialized maturation. Therefore, in regions that showed a Category × Age interaction, we examined the simple effect (Keppel and Zedeck 1989) of age group for FSI_B_ and OSI_B_ separately. If the simple effect of age was significant only for FSI_B_ (and if FSI_B_ increased with age) then specialized maturation would be supported. If the simple effect of age was significant for both FSI_B_ and OSI_B_ (and if FSI_B_ showed an increase but OSI_B_ showed a decrease with age), then competitive interactions would be supported. Results are summarized in Table 1.

**Figure 4:**
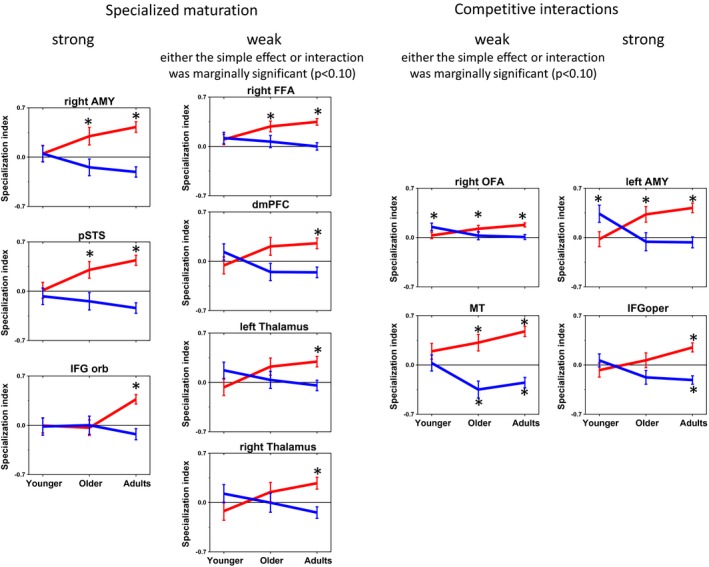
Developmental trajectories in ROIs (regions of interest). ROIs with profiles of specialized maturation or competitive interactions are shown “Strong” profiles mean that the Age × Category interaction was significant and the simple effect(s) or interest were also significant. “Weak” profiles mean that either the Age × Category interaction or the simple effect(s) or interest were marginally significant * indicates that FSI_B_ or OSI_B_ was significantly different from 0 for the given age group according the a one‐sample *t*‐test. Error bars are standard error.

Of the 14 regions, 11 regions showed a significant or marginally significant Age × Category interaction. Bilateral occipital poles and the left OFA did not show an interaction so no simple effects analyses were conducted in these regions. However, simple effects analyses conducted in the other 11 regions provided evidence for both specialized maturation and competitive interactions (Fig. 4). The right pSTS, right AMG and right IFG‐pars orbitalis all showed evidence for specialized maturation in that the Category × Age interaction was significant and the simple effect of age was only significant for FSI_B,_ indicating increased tuning to faces with age but not objects. The right FFA, dmPFC, and bilateral thalamus showed weak patterns of specialized maturation, because either the simple effect of age for FSI_B_ or the interaction was marginally significant. Competitive interactions emerged in the right IFG‐ pars opercularis and left AMG, in that the simple effect of age was significant for both FSI_B_ and OSI_B_ and these age trends were in opposite directions, indicating increased tuning to faces and increased pruning of responses to objects. A weaker form of competitive interactions emerged in the right OFA and right MT, because one or two of the simple effects was marginally significant.

Another analysis was conducted to confirm that the regions showing competitive interactions had a different age profile of object‐specialization than regions showing specialized maturation, as the profile for object‐specialization is what differentiates the two accounts. OSI_B_ was averaged in the seven regions that showed specialized maturation and in the four regions that showed competitive interactions to yield two OSI_B_ values per subject. These values were then submitted to an ANOVA with OSI_B_ as the dependent variable, profile type (competitive interaction, specialization maturation) as the repeated factor and age (adult, older, younger) as the between‐groups factor. If the two profile types are indeed different in terms of object specialization, then the Age × Profile interaction should be significant and the simple effect of age should only be significant for the competitive interactions profile. This was confirmed with a significant Age × Profile interaction, *F*(2, 88) = 3.2, *P* = 0.046, and a simple effect of age only for the competitive interaction profile, *F*(2, 88) = 6.9, *P* = 0.002, but not for specialized maturation, *F*(2, 88) = 1.5, *P* = 0.226.

Although a significant age effect for either category or both categories reflects developmental change, it is not clear at which age specialization emerges. Potentially, this could be tested by conducting post hoc *t*‐tests between age groups to determine whether children show lower (or higher) specialization than adults. However, these post hoc comparisons could reveal significant age differences, even if face or object specialization itself was not very pronounced. In other words, it would be important to determine whether FSI_B_ and OSI_B_ were different from 0 at any age because that would indicate that significant face or object specialization emerged at that age. Therefore, for each significant simple effect of age (for FSI_B_ or OSI_B_ or both) we conducted a one‐sample *t*‐test against 0 for each age group separately. For specialized maturation, these *t*‐tests were only conducted for FSI_B_; for competitive interactions, these *t*‐tests were conducted for both FSI_B_ and OSI_B_ (given that these were the significant simple effects of age). An early developmental process would be indicated if younger children's FSI_B_ or OSI_B_ showed a significant deviation from 0. A late developmental process would be indicated if only adult's FSI_B_ or OSI_B_ significantly deviated from 0. The ages at which the FSI_B_ or OSI_B_ deviated from 0 is indicated by asterisks in Figure 4. For specialized maturation regions, the bilateral thalamus, dmPFC and right IFG‐orbitalis showed significant face specialization only in adults indicating later developmental specialization, whereas in the right FFA, right pSTS, and right AMG, significant face specialization was present for older, but not younger children indicating earlier specialization occurring sometime before age 9. For regions showing competitive interactions, the left AMG, right OFA and right MT showed significant face specialization for older children and adults and the right IFG pars opercularis showed significant face specialization only for adults. Right OFA and left AMG also showed significant object specialization only for younger children. Results were similar using only half of the sample, with only a few exceptions (see Appendix S2).

Although FSI_B_ and OSI_B_ served as the primary specialization indices, the results for the other approaches to calculating face and object specialization are presented in Appendix S2. The main effects and interactions results are somewhat similar across measures, especially between FSI_A_ and FSI_B_. But note that FSI_B_ measure was chosen based on its distributional properties and greater face validity and not based on the significance of results from the repeated measures ANOVAs.

The one‐sample *t*‐test to analyze whether percent signal change was different from 0 for the face condition revealed that fMRI signal was different for adults in all regions (*P* < 0.05). For older children, fMRI signal for faces was different from 0 in all regions (*P* < 0.05), except the IFG‐orbital, right thalamus, and right MT. For younger children, fMRI signal for faces was different from 0 in the right FFA, right and left OFA, the right IFG‐opercular and right IFG‐orbital cortex (*P* < 0.05), but not in left and right AMG, left and right thalamus, dmPFC, MT or the pSTS, the IFG‐orbital, right thalamus, and right MT.

### Results for the analysis of FFA size

Other studies have reported that the right FFA increases in size with age (Golarai et al. 2007, 2010; Scherf et al. 2007; Peelen et al. 2009; Haist et al. 2013) and the results in Figure 3 also indicate that this is also the case when the same threshold is applied to all age groups (in this case, *P* = 0.005, uncorrected). Consequently, the failure to find face specialization in the youngest children in this study may be due to using a larger FFA (as defined in adults) which may have included many voxels that were not specialized for faces in children. In other words, if a smaller FFA (i.e., roughly the same size as the FFA shown in Figure 3 for younger children) had been applied to the child data, the FSI_B_ may be comparable to that of adults, or it might be significantly different from 0, because it would only include the most face specialized voxels in younger children. We also examined whether a larger FFA than used in the primary analysis would dilute FSI_B_ in any age group to further determine whether face specialization depends on spatial extent of activation, more generally.

**Figure 5:**
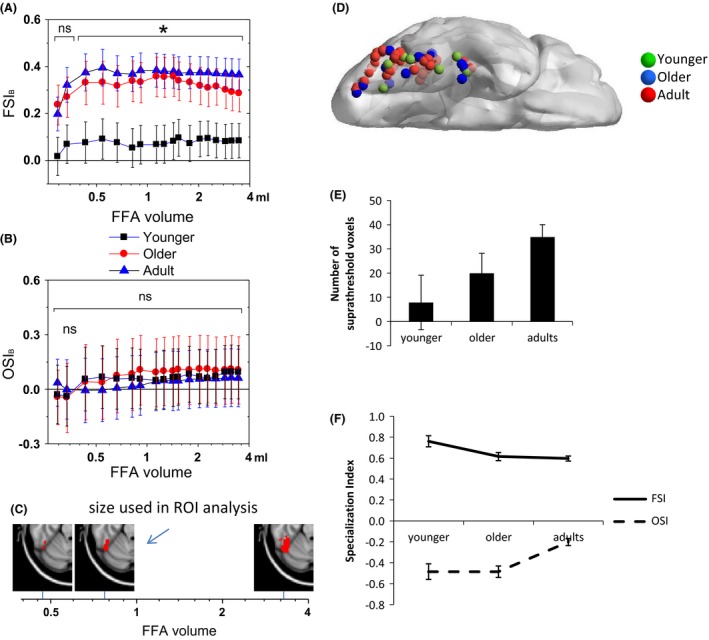
(A) FSI_B_ (Face specialization index) as a function of FFA (fusiform face area) volume in each age group. The * indicates that the main effect of age was significant at each of the volumes greater than .43 mL. All FSI_B_ values for adults and older children were significantly greater than 0 according to one‐sample *t*‐tests. (B) OSI_B_ (Object specialization index) as a function of FFA volume in each age group. None of the OSI age effects was significant. Error bars are standard error of the mean. (C) Illustration of different FFA volume sizes. (D) Illustration of the location of the peak face‐preferential voxel in each subject who showed one or more voxel in the anatomically defined fusiform gyrus at an uncorrected *P* < 0.001. (E) The average number of suprathreshold face‐preferential voxels in the fusiform gyrus by age group. (F) FSI_B_ and OSI_B_ calculated in all suprathreshold voxels as a function of age group (*n* = 8 younger children, *n* = 15 older children and *n* = 39 adults). All specialization indices were different from 0 according to a one‐sample *t*‐test.

To test these possibilities, we examined age effects on FSI_B_ (and OSI_B_) as a function of FFA volume (Fig. 5). For FSI_B_, age group effects were significant at each FFA volume greater than 0.43 mL (*P* < 0.05) with adults showing a higher FSI_B_ than younger children but not older children. For smaller FFA volumes (*z* > 2.9, size < 54 voxel or 0.43 mL), the age effect was no longer significant. However, one‐sample *t*‐tests that examined whether FSI_B_ was different from 0 at each FFA volume for each age group revealed that FSI_B_ was different from 0 at all volumes for adults and older children but was not different from 0 at any volume for younger children. OSI_B_ was not significantly different from zero at any size for any age groups. Therefore, the present findings related to increased FSI_B_ with age (at least in the right FFA) are not driven by arbitrary thresholding or activation extent because they are consistent across different levels of thresholding, with the exception of very small volumes. OSI_B_ did not show any age differences as a function of FFA size.

### Individual‐subject face‐preferential responses

The percent of subjects that showed surviving face preferential voxels in the anatomically defined fusiform gyrus was 76% of adults, 63% of older children and 50% of younger children. These voxels were scattered throughout the posterior, mid‐ and anterior fusiform gyrus (Fig. 5D). The effect of age group on number of surviving voxels in the fusiform was marginally significant, *F*(2, 61) = 2.99, *P* = 0.058 (Fig. 5E). Post hoc comparisons using Tamhane's *t*‐test indicated that adults had more surviving voxels than younger (*P* = 0.001) but not older (*P* = 0.33) children. Among subjects with suprathreshold voxels, the majority of adults (74%) had 10 or more suprathreshold voxels, whereas only half of older children (53%) and only 12% (1 out of 8) of younger children had 10 or more surviving voxels. Interestingly, the effect of age on the anterior‐posterior locus of the peak voxel in the fusiform was marginally significant, *F*(2, 61) = 2.66, *P* = 0.079, as was the effect of age on the dorsal‐ventral locus of the peak voxel: *F*(2, 61) = 3.1, *P* = 0.053. Children activated a more anterior and ventral aspect of the fusiform than did adults; however, post hoc comparisons indicated no significant differences. In addition, among subjects that showed suprathreshold activation, the Category × Age repeated measures ANOVA revealed a significant interaction, *F*(2, 59) = 11.8, *P* < 0.0001. The simple effect of age on FSI_B_ was significant, *F*(2, 61) = 3.98, *P* = 0.024. Younger children showed a higher FSI_B_ (*P* = 0.0001) than adults (Fig. 5F). The effect of OSI_B_ was also significant, *F*(2, 61) = 13.04, *P* = 0.0001. In this case, adults showed a lower OSI_B_ than older children (*P* = 0.002). To explore whether some of these age group differences in various aspects of activation (number of voxels, locus or degree of face and object specialization) reflect a developmental change (rather than some other individual difference), we conducted Spearman rank correlations with age among children. However, none of these features of activation was correlated with age.

## Discussion

One of the primary goals of this study was to determine whether children show the same degree of face specialization as adults in brain regions recruited by adults for face processing. Several different types of analyses converged on the finding that younger children do not show the same degree of face specialization as adults. This was demonstrated by lower face specialization indices in younger children, no activation that survived statistical thresholds in the voxel‐wise group analyses in younger children, fewer younger children who show supra‐threshold voxels when individual‐subject ROI were examined, and fewer surviving voxels in younger children's fusiform gyrus ROIs.

Also, face specialization increased with age in many critical components of the face network, in agreement with other studies (Aylward et al. 2005; Golarai et al. 2007; Peelen et al. 2009; Joseph et al. 2011, 2015b). Face specialization was present in older children in the majority of regions (right FFA, right AMG, left AMG, right pSTS, right MT and right OFA) but did not emerge until young adulthood in frontal regions (right dmPFC, right IFG‐orbital, right IFG‐opercular) and the thalamus. The finding of delayed specialization in frontal regions is not surprising given the protracted development of these regions (Paus 2005). Importantly, the face‐specialization index not only assessed degree of face preference relative to nonface categories but also controlled for age‐related differences in activation magnitude. Therefore, the developmental changes in the present study were scaled to the maximum level of activation in a region for each individual. Moreover, the lack of face specialization in younger children was not driven by failure to activate some of the core face network regions because younger children showed an fMRI signal that was greater than baseline in the right FFA, bilateral OFA and the two inferior frontal regions. However, these regions were not more strongly activated for faces compared to the other two experimental conditions, as was the case in adults. In addition, degree of face specialization was not dependent on the volume of the group‐defined FFA (see Section Regions of interest results). The majority of the findings in the present paper support the idea of minimal face specialization in younger children, as a group, but a subset of children showed face specialization in the fusiform gyrus, which is discussed more in Section Competitive interactions.

Another major goal was to determine whether regions that showed increased face specialization with age had concomitant decreases in object specialization, in support of competitive interactions, or whether increased face specialization emerged with no change in object specialization with age, in support of specialized maturation. The present study provided evidence for both specialized maturation and competitive interactions in the development of functional properties of adult face network regions. The evidence for each of these frameworks is discussed in turn below.

### Specialized maturation

Within the developmental time window examined here, several regions primarily in the right hemisphere showed increased face specialization with age, but no changes in object specialization with age – the right FFA, right pSTS, dmPFC, right AMG, right IFG‐orbitalis and bilateral thalamus. Hence, cortical specialization for faces emerges gradually in these regions, but not by competing with object representations. In one sense, specialized maturation could indicate a developmental process that unfolds gradually over time and is immune to functional reorganization with development, as predicted by the Maturational viewpoint (as discussed in Johnson 2005; Joseph et al. 2011). The strong form of this framework suggests that the specialized function of a particular brain region is determined at birth, and others have made a similar argument with respect to face processing (Farah et al. 2000). However, face specialization did not emerge until adulthood in the frontal regions and the bilateral thalamus and face specialization was present in older children in the right AMG, right FFA, right pSTS. Neither of these outcomes supports the strong form of specialized maturation, but they do support the constructivist viewpoint that tuning to faces increases with age, but not by competing with responses to nonfaces.

Another consideration regarding the interpretation of specialized maturation is that this particular maturational profile may only hold within the developmental time window examined here. It is entirely possible that in an earlier time window, evidence for competitive interactions would emerge in that regions that showed face‐specialized maturation may show object specialization at an earlier age than tested in this study. Cantlon et al. (2011) tested face‐specialization in the right FFA (compared to responses to other visual categories) in 4‐to‐5 year old children and showed a face‐preferential response even at this early age, rather than a preference for nonfaces, which would support specialized maturation of this region in an earlier time window. However, they also showed that face identification accuracy in children was correlated with a lower right FFA response to nonfaces (letters), but was not correlated with the right FFA response to faces. They interpreted this finding as a marker of pruning, or the attrition of responses to nonpreferred categories. In the present conceptualization this would be consistent with competitive interactions. Consequently, specialized maturation of the right FFA observed within the developmental time window of this study may have been preceded by competitive interactions earlier in development. However, without specifically testing earlier developmental windows using the present analytic approach, we cannot rule out that competitive interactions emerge earlier in development in the right FFA.

Interestingly, most of the regions that showed specialized maturation during childhood (right pSTS, right FFA, right IFG orbitalis and right dmPFC) overlap with regions involved in social cognition and mentalizing. For example, the pSTS/TPJ (temporo‐parietal junction) is a central locus in processing the mental states of others (Saxe and Powell 2006). The pSTS and right IFG orbitalis are associated with perceptions of trustworthiness in faces (Verosky and Todorov 2010) and inferring feelings or mental states of others from facial information (Moor et al. 2012; Spunt and Lieberman 2012; von dem Hagen et al. 2013; Johnston et al. 2013). The regions showing specialized maturation are also reported to undergo developmental change. For example, the right FFA continues to develop throughout adolescence (Golarai et al. 2007; Passarotti et al. 2007; Peelen et al. 2009) and the right IFG is reported to have similar developmental trajectories as the fusiform gyrus, in terms of signal change or activation ratio (Shaw et al. 2012). Functional development of the STS has also been reported (Moor et al. 2012) (but see Golarai et al. (2007) who reported no differences across ages in the size of the STS face‐selective region). Hence, this study's findings of increased face specialization with age in these regions are consistent with other findings of development of these regions. The present study additionally showed that none of these regions exhibited face specialization at the youngest age (5–8 years).

### Competitive interactions

Competitive interactions emerged in the left AMG, right MT, right IFG pars opercularis and right OFA. In each of these regions face specialization increased with age while object specialization decreased with age. Although these regions are often reported in face processing tasks, it is interesting to note that they are not typically or as strongly associated with social information processing, like the specialized maturation regions described above are. In fact, these regions are characterized by their participation in more general perceptual or cognitive functions that may not be face‐specific. The AMG, for example, has been described as part of a salience detection system that is rapidly engaged for any salient or important stimulus (Ohman et al. 2007). Likewise, although the right OFA is an integral part of the face network, and receives feed‐forward and re‐entrant feedback from face‐sensitive areas, including the FFA (Kadosh et al. 2011), the OFA has been recently described as involved in making fine perceptual discriminations of visually homogenous categories other than faces (Haist et al. 2010; Collins et al. 2012). The precise function of OFA is still not clear as it seems involved in the processing of different face properties (Maurer et al. 2002; Pitcher et al. 2011) and both early (Pitcher et al. 2007) and later face processing stages (Rotshtein et al. 2007). Nevertheless, the OFA does seem more strongly implicated in perceptual processes that are not necessarily face‐specific.

The regions showing competitive interactions are also reported to undergo developmental change. Although age group comparisons of AMG activation have yielded mixed findings as to whether children and adolescents show less, more or comparable levels of activation compared to adults (Lobaugh et al. 2006; Killgore and Yurgelun‐Todd 2007; Guyer et al. 2008; Hoehl et al. 2010; Vasa et al. 2011; Ebner et al. 2013) studies that have examined correlations with age over a developmental time window (as opposed to group comparisons of adults versus children or adolescents) also report that AMG activation for neutral faces (Joseph et al. 2015b) or emotional faces versus scrambled images increases with age (Todd et al. 2011; Pagliaccio et al. 2013). Similarly, the present study showed increased face specialization with age in the left AMG. The right OFA also undergoes developmental change (Joseph et al. 2011). Joseph et al. (2012) conducted a functional connectivity analysis using graph‐theory and showed that connectivity of the right OFA changes not only during childhood but also from childhood to adulthood. Specifically, the right FFA and right OFA coalesced into the same module during childhood, but were dissociated into different modules by adulthood. Hence, the right OFA and AMG tend to show fairly dynamic changes in functionality during childhood, which is consistent with the present finding that these regions shift from object to face specialization during development.

### Potential interactions among face network components

Johnson (2011) has suggested that the increased specialization of function during development is accomplished by the interaction or connectivity patterns among brain regions. As an example, increased tuning in receptive fields and synaptic pruning in surrounding cortical areas is one potential developmental mechanism that may lead to increased cortical specialization of function with age. Although the study did not address connectivity or interactions among brain regions directly, one potentially interesting finding in this study relevant to this point is that profiles of specialized maturation and competitive interactions were often juxtaposed in spatially proximal brain regions. As outlined in Table 2, these couplings were observed in occipito‐temporal, lateral temporal and inferior frontal cortex. In the occipito‐temporal cortex, the right FFA showed (weak) evidence for specialized maturation whereas right OFA showed (weak) evidence for competitive interactions. As described above, the right OFA may subserve early perceptual processes that differentiate visually similar items from each other (Haist et al. 2010; Collins et al. 2012) or process more elemental features (Maurer et al. 2002; Pitcher et al. 2011). These different types of processing are not necessarily face‐specific, but are likely essential to engage this processing for some face tasks. The right FFA has been attributed with more face‐specific processing such as identifying specific persons, which relies on being able to make fine distinctions among individual faces, a process that also engages the OFA, but not exclusively for faces (Haist et al. 2010; Collins et al. 2012). Of note, this same capacity for fine perceptual differentiation is a component process of perceptual expertise. Another possible function of the right FFA is to integrate features into a unified face percept (Nichols et al. 2010; Collins et al. 2012). This integrative process may depend on input about individual face features from the OFA (Pitcher et al. 2007). Although the exact function of the right FFA is debated, we suggest that as a person matures, the increased tuning to faces in the right FFA may depend on co‐opting the perceptual processing engaged in the right OFA. More specifically, in order to identify faces at an expert level (as typical adults do), the right FFA may depend on the process of perceptual differentiation in the right OFA. Children may lack the synergistic coupling between these two regions (see Cohen Kadosh et al. (2011)). Similarly, if the right FFA is involved in integrating face features into a unified percept, the right OFA may provide critical input of processing face features (Pitcher et al. 2007). In fact, (Joseph et al. 2015a) recently reported that integrating face features into a holistic face percept may not emerge until adulthood. Therefore, the right OFA would not be recruited preferentially for processing face features in children if there is no need to integrate those features in the right FFA. These speculations on the development of coupled processing of the right OFA and right FFA will need to be tested in future studies, preferably with effective connectivity analysis.

**Table 2:** Summary of development profiles in nearby brain regions

	Coupled regions	Maturational profile	Proposed function (s)
Occipital‐temporal regions	Right occipital face area	Competitive Interactions	a ‐ Differentiating items from visually homogenous categories Collins et al. (2012); Haist et al. (2010)
			b ‐ Processing face features Pitcher et al. (2007); Nichols et al. (2010)
	Right fusiform face area	Specialized maturation	a ‐ Face identification Haxby et al. (2000); Kanwisher and Yovel (2006)
			b – Integrating features into a unified face percept Collins et al. (2012); Nichols et al. (2010)
Temporal regions	Right middle temporal area	Competitive interactions	Visual motion processing Tootell et al. (1995)
	Right posterior STS	Specialized maturation	Perceiving the changeable aspects of faces Haxby et al. (2000)
IFG	Right IFG opercularis	Competitive interactions	Motor mirroring Johnston et al. (2013)
	Right IFG orbitalis	Specialized maturation	Reading mental states from Faces Moor et al. (2012)

IFG, inferior frontal gyrus.

Another similar coupling of maturational profiles was with the two regions located in right posterior lateral temporal cortex, the pSTS and MT, which showed specialized maturation and competitive interactions, respectively. Area MT is considered the primary site for processing visual motion (Tootell et al. 1995), whereas Haxby et al. (2000) has suggested that pSTS is involved in perceiving the changeable aspects of faces, a higher order abstraction that relies on understanding biological motion. In fact, pSTS is heavily involved both in perceiving biological motion (Grossman et al. 2005), but also in perceiving eye gaze or other socially relevant information (Pierce and Redcay 2008). Again, the more basic perceptual processing region MT is not necessarily face‐specific, but this region can be recruited for face‐specific processing (Miki and Kakigi 2014; Rossi et al. 2014) and we suggest that it becomes increasingly more strongly co‐opted for face tasks with development.

Finally, two frontal regions, the right IFG‐orbitalis and right IFG‐opercularis, showed a similar coupling. Johnston et al. (2013) suggested that the right IFG‐orbitalis is involved in active motor mirroring or imitation in contrast with IFG‐opercularis which is more involved in passive motor mirroring. In addition, Spunt and Lieberman (2012) showed that the IFG‐orbitalis was involved in attributing reasons for why an actor was displaying emotions whereas the IFG‐opercularis was involved in determining which facial features were being used to display an emotion. Others have also reported that the IFG‐opercularis is involved in executing and perceiving facial expressions (Carr et al. 2003; Dapretto et al. 2006). In both of these examples, the IFG‐orbitalis is involved in a higher level of processing social information, related to taking another person's perspective in order to accomplish the task at hand. The IFG‐opercularis is involved in the more basic function of motor mirroring that is not necessarily face‐specific.

Although we did not find the same pattern of spatially proximal activation in the AMG or thalamus, the right AMG and posterior bilateral thalamus showed specialized maturation of faces. Some have suggested that the AMG may automatically process faces (Winston et al. 2002). In addition, Dyck et al. (2011) recently suggested that the right AMG is involved in automatic emotional responses to faces whereas the left AMG (which showed competitive interactions in the present study) is more involved in intentional mood control, suggesting a more generalized role for the left AMG. The thalamus activation in the study was consistent with the location of the pulvinar nucleus. Nguyen et al. (2013) reported that the pulvinar contains cells that respond very rapidly (within 50 msec) to face‐like patterns in nonhuman primates. Taken together, these findings suggest that the right AMG and bilateral thalamus may be involved in rapid, automatic face detection, even in younger children, but as individuals mature, these regions become even more tuned specifically to faces.

In sum, the regions that show competitive interactions during the development of face specialization in this study are implicated in particular perceptual or cognitive functions that apply not only to faces, but to other categories as well. Presumably, these functions are co‐opted for faces as an individual develops and motivation toward social stimuli increases (Scherf et al. 2012), as outlined in Table 2. The later maturation of face specialization in these regions may support a role in continuously optimizing face processing performance after childhood. We do not suggest, however, that these regions are necessarily permanently co‐opted for face processing in the adult, so that they become dedicated only to faces; rather, they are likely recruited more strongly for face‐specific processing given specific tasks demands in order to achieve high levels of expertise with processing faces.

### Development of the right FFA

The present study showed that the right FFA is among those ROIs that developed early, in that FSI_B_ was significantly different from 0 in older children and adults (whereas some ROIs only showed a significant FSI_B_ in adults). Cantlon et al. (2011) also reported face specialization in the right FFA very early in development. Haist et al. (2013) reported no increase in signal magnitude in the right FFA from age 6 to 16, also suggesting early development of the right FFA (but FFA volume did increase in that study). Joseph et al. (2011) reported an increase in face specialization with age in the right FFA, but this region did not show the most pronounced developmental change: other visual areas, including the left FFA and right OFA showed stronger increases in face specialization with age. A recent study (Joseph et al. 2015b) examined developmental changes in face specialization using the same FSI as in the present study from 6 to 17 years of age. Face specialization increased significantly with age in the left FFA but not in the right FFA. Conversely, some prior studies have reported that the FFA continues to develop throughout adolescence (Golarai et al. 2007; Passarotti et al. 2007; Peelen et al. 2009). In general, the right FFA does appear to show increases in face specialization with age, but in some studies these changes were modest compared to other brain regions.

An important consideration about the degree of face‐specialization in the fusiform gyrus is that there is individual variability in the location of the FFA (Saxe et al. 2006). For this reason, some studies in adults prefer to analyze individual‐subject face preferential responses rather than use group‐defined ROIs. This is also an important consideration in developmental studies because face network organization can shift and change with age. If, in fact, the location of the “FFA” changes with age, the group‐defined ROI approach may underestimate the degree of face specialization in children. This is an important issue, but we note that it asks a different question than the primary question in this study, which was: Do children show the same degree of face specialization as adults show in regions that are recruited by adults during face viewing? The answer to this question is no, at least with respect to younger children, as discussed at length above. However, the analysis of individual‐subject face‐preferential responses addresses a separate, but important question: Do children show face preferential responses in a different location than adults do? Although the group maps in Figure 3 indicate that younger children only show face‐preferential activation at an uncorrected level, we further addressed whether younger children show face‐preferential activation in a different location in the right fusiform gyrus by analyzing individual‐subject face‐preferential responses. This analysis revealed that some children show a strong face preference in the fusiform gyrus, even stronger than that of adults when all suprathreshold voxels were considered for a subject. However, younger children activated fewer face‐preferential voxels than adults and only 1 of the younger children activated more than 10 voxels in the fusiform gyrus, compared to the majority of adults who activated 10 or more. Also, the locus of the peak face‐preferential response was more anterior than that of adults and the locus did not correlate with age in children. In summary, the analysis of individual‐subject face‐preferential responses indicates that some children do exhibit strong face specialized responses, albeit in a more anterior locus than adults. However, the primary analysis in this study indicates that as a group, children do not show the same degree of face preference as adults in the fusiform gyrus, but some individual children do show a face preference.

### Developmental changes in the functional organization for face processing

Although many studies on the development of face processing have focused on specific functional regions (FFA, OFA, STS, and parahippocampal place area), some studies have examined whole‐brain networks (Haist et al. 2013, 2010; Joseph et al. 2011; Passarotti et al. 2003). In general, when whole‐brain activation patterns are considered, children show more diffuse (Passarotti et al. 2003), more extensive (Haist et al. 2013) or qualitatively different (Joseph et al. 2011) patterns of activation compared to adults. The specific regions that are recruited differently in children than adults vary widely across studies, and no consistent pattern has emerged. This inconsistency could be due to the different statistical contrasts used, which may highlight different demands on perceptual differentiation. For example, Joseph et al. (2011) and Haist et al. (2013) contrasted faces with objects that had three‐dimensional structure, and reported more additional regions of activation in children versus adults. In contrast, this study and Passarotti et al. (2003) study, which contrasted faces versus textures, reported fewer additional regions of activation in children. Another reason for inconsistent regional activation patterns across studies may be the age windows examined. In general, as Joseph et al. (2011) suggested, children over age 8 show activation patterns that are more consistent with adults than do children under age 8. This study confirmed this general trend. The developmental period from 8 to 10 years of age may represent an important transitional period for the development of face specialized responses, based on behavioral findings (Carey and Diamond 1994; Mondloch et al. 2002; McKone et al. 2012). In addition, electrophysiological findings suggest that from 8 to 10 years of age, there is a marked decreased in latency of the face‐specific N170 but latency remained fairly stable after age 10 (Taylor et al. 1999; Itier and Taylor 2004). Consequently, this age range may represent a particularly dynamic period of functional brain reorganization to support higher level face functions of decoding emotions and social cognition. In complex and dynamic systems, periods of transition are marked by greater variability as the system reorganizes (Smith and Thelen 2003). Hence, the regional differences found across studies may reflect greater variability in brain‐to‐function mappings as the system reorganizes (Scherf et al. 2012).

### Limitations

One potential limitation of this study was that we did not sample a continuous age range throughout childhood and adolescence. Although there were clear developmental differences detected within this time window of 5–12 years, a more rigorous test of the maturational and IS accounts would involve examining ages much younger (toddler or preschool ages) or older (adolescents) than tested here. For example, in regions that showed specialized maturation, it is possible that a pruning process occurs before age 5. Similarly, some face‐related processing shows nonlinear development from childhood to adolescence (Scherf et al. 2011), so regions that appear to be specialized for faces in older children may regress in adolescence. However, Joseph et al. (2015a) used similar methods as used in the present study and examined face specialization from age 6 through 18. That study showed significant increases in face specialization with age that appeared to be linear through the adolescent years; however, linear versus nonlinear trends were not tested in that study. Therefore, it would be important for future studies to characterize such developmental trajectories as a step toward elucidating mechanisms of change and plasticity for specialized cognitive capacities.

Another potential limitation of this study was the use of a passive viewing task, which may not be ideal for revealing neurodevelopmental changes in face processing. The advantage of using a passive viewing task is that it is simple enough even for the youngest subjects, thereby controlling for demands on cognitive processing across age groups. However, passive viewing does not necessarily capture the relevant perceptual and cognitive processes that are known to change with age, such as configural versus analytic face processing (Diamond and Carey 1977) or decoding emotion in faces (Todd et al. 2011; Marusak et al. 2013). In this case, then, the present task may have underestimated maturational changes. A task that required active processing of the stimuli might engage face processing areas more in children. Consequently, future studies should address whether the maturational changes observed here with passive viewing apply to paradigms that require more active processing of faces.

Another limitation was that, in some cases, the maturational profiles of specialized maturation and competitive interactions were only weakly supported, indicated by either a marginally significant interaction or simple effect, or both. These marginal effects could be driven by higher variability in small samples. Indeed, when only half of the adult subjects were analyzed, some of the significant interactions and simple effects obtained with the full adult sample became marginally significant or not significant (Appendix S2). However, in this case, the adult means remained quite stable when the adult sample was half the size. This does not necessarily guarantee that the means for children would also remain stable, however. Different rates of maturation and different inherent face capabilities can have a significant impact on brain response to faces. In fact, the analysis of individual‐subject face preferential activation peaks indicates significant variability across subjects. Therefore, the ideas of specialized maturation and competitive interactions should be tested in larger samples of children in future studies.

## Conclusion

The present study provided evidence for increased tuning of face responses during development with or without pruning of nonface responses. The majority of neuroimaging studies of typical face development have focused primarily on tuning processes. However, in order to examine pruning processes, which is a significant developmental event, studies should focus on both face and nonface responses in an extended network of brain regions. The primary finding from this study was that regions associated with higher level face capacities like social cognition showed stronger evidence for specialized maturation, or increased tuning for faces with age but no change in object tuning. In contrast, regions associated with more basic perceptual functions like detecting salient stimuli, processing visually similar categories (i.e. perceptual differentiation), visual motion and motor mirroring showed evidence for competitive interactions; that is, increased tuning for faces occurred in parallel with greater pruning of nonface responses. Interestingly, these developmental profiles of specialized maturation and competitive interactions are often co‐localized in spatially contiguous areas of cortex, suggesting that the more basic perceptual processes may provide essential input into the more face‐dedicated brain regions. However, early in development, these regions are not recruited more strongly during face processing. The coupled recruitment of these regions may thus increase with age, thereby supporting increased expertise for faces with development.

## Conflict of Interest

None declared.

## Supporting information


**Appendix S1.** Comparison of approaches to assess specialization.
**Appendix S2.** Results from the repeated measures ANOVAs conducted using different specialization.
