# Supplementary material for: Pruning or tuning? Maturational profiles of face specialization during typical development
Source: Brain Behav. 2016 Apr 15;6(6):e00464. doi: 10.1002/brb3.464 (PMC4907975; doi:10.1002/brb3.464)
Supplement: Supplementary file 1 — Appendix S1. Comparison of approaches to assess specialization. Appendix S2. Results from the repeated measures ANOVAs conducted using different specialization. [file BRB3-6-e00464-s001.pdf]

Appendix A

Comparison of approaches to assess specialization

Several prior studies have addressed the issue of defining specialization of the fMRI response for a particular visual category relative to other visual categories. In some of these studies, the definition of specialization depends on a set of statistical comparisons of the fMRI signal across different experimental conditions (Joseph et al., 2002, Joseph and Gathers, 2003, Gathers et al., 2004, Joseph et al., 2006a, Joseph et al., 2011) whereas others have combined fMRI signal from different experimental conditions into a “specialization index” (Golarai et al., 2007, Simmons et al., 2007, Joseph et al., 2011). With respect to the statistical comparison approach, Joseph and colleagues offered several definitions of specialization that vary in degree of stringency. For example, the most stringent definition of specialization requires that the category of interest must produce a statistically greater signal than all other experimental conditions (including the baseline condition) and none of the experimental conditions can be different from each other. This is referred to as a “selective” response because only the category of interest activates a particular region or voxel. The least stringent definition of specialization only requires that the category of interest yields a statistically greater signal than the baseline condition and at least one other condition. This is referred to as a “preferential” response because the category of interest is preferred over some other categories and conditions, but the response is not exclusive to the category of interest. Other definitions of specialization yield an intermediate degree of selectivity. Joseph and Gathers (2002) reported that regions like the FFA do not yield a selective response, but instead, are associated with a less stringent profile of specialization. However, we use the term “specialization” in this paper.

We examined the distributional properties of different indices that describe the degree of specialization for faces or objects. For each index described below, we extracted fMRI signal change within regions of interest (ROIs) that were reported by Collins et al. (2012) as face-preferential regions. We used ROIs defined by Collins et al. so that the ROIs could be defined in an independent manner from the way they are defined in the present study. Percent signal change for faces, objects and textures (or raw intensity values for these conditions, as used for one of the indices below) were then extracted within these ROIs and used to create different specialization indices using the present dataset because this dataset has more subjects than the Collins et al. dataset and includes children so that the distributional properties could be examined in adults and children.

With respect to the specialization index approach, Golarai et al. (2007) used the following formula to define degree of face specialization (FSI, face specialization index):

$$\mathrm{FSI}_{\mathrm{Golarai}} = \frac{Face_{pc} - Object_{pc}}{Face_{pc} + Object_{pc}}$$

Where F_{pc} and O_{pc} are percent signal change for faces or objects, respectively, relative to baseline.

But Simmons et al. (2007) noted that this particular formula can yield extremely high values if negative values are in the formula so they suggested a correction for negative values as:

$$\mathrm{FSI}_{Simmons} = \frac{Face_{adj} - \ Object_{adj}}{Face_{adj} + \ Object_{adj}}$$

Where

$$Face_{adj} = F_{pc} + \ \left|minimum(F_{pc}, O_{pc,} \, T_{pc}, 0)\right|$$

$$Object_{adj} = O_{pc} + \left|minimum(F_{pc}, O_{pc,} T_{pc}, 0)\right|$$

Here, if percent signal change is negative for any of the conditions, the absolute value of the most negative value is added to each condition. If all percent signal changes are positive, no adjustments are made.

Joseph et al. (2011) did not use percent signal change in their specialization index formula, but instead used the intensity values from all experimental conditions and baseline in order to avoid the problem of negative values. In addition, they used two control conditions (natural objects, manufactured objects) and a baseline condition (rest) and the formulas above only include a condition of interest and one control condition so their formula incorporated all experimental conditions:

$$\mathrm{FSI}_{\mathrm{Joseph,2011}} = \frac{F_i - (M_i + N_i + R_i)/3}{(M_i + N_i + R_i)/3}$$

Where F_i , M_i , N_i , R_i are the intensity values for faces, manufactured objects, natural objects and rest, respectively.

Because the present study uses a condition of interest (e.g., face) and two control conditions (e.g., object, texture) plus resting baseline, like Joseph et al. (2011), we explored several FSI formulas. In the first FSI, we modify the formula used by Joseph et al. (2011) to accommodate the conditions in the present study:

$$\mathrm{FSI}_{JGB} = \frac{F_i - (O_i + T_i + R_i)/3}{(O_i + T_i + R_i)/3}$$

Where F_i , O_i , T_i , R_i are the intensity values for faces, objects, textures and rest, respectively. An object specialization index was also computed where the F_i and O_i terms were

switched. The distributions of FSI_{JGB} and OSI_{JGB} for children and adults (over all subjects and all regions) are shown in Figures A1 and A2. The distribution of FSI_{JGB} is positively skewed in adults with more extreme positive values than in children. This is expected because the ROIs were defined as face-preferential at the voxel level so the FSI would naturally indicate a greater preference for faces. The OSI_{JGB} distributions are not skewed in either children or adults.

In the next FSI, percent signal change relative to fixation baseline is used rather than signal intensity values and the denominator is the range of percent signal change values in a given region for a given subject across all three conditions:

$$\mathrm{FSI}_A = \frac{F_{pc} - (O_{pc} + T_{pc})/2}{\mathrm{Max}(F_{pc}, O_{pc}, T_{pc}) - \mathrm{Min}(F_{pc}, O_{pc}, T_{pc}, 0)}$$

and F_{pc}, O_{pc} , T_{pc} are percent signal change for faces, objects, or textures, respectively, relative to baseline. The distribution of FSI_A in children and adults is shown in Figures A1 and A2. These distributions are not as kurtotic as those for FSI_{JGB} and OSI_{JGB} and the FSI_A distribution is now negatively skewed in adults and the OSI_A distribution is negatively skewed in children.

We also explored another formula that uses a modification of the Simmons et al. adjustment and scales the differential response only to the maximum value (rather than the range):

$$\mathrm{FSI}_B = \frac{Face_{adj} - (Object_{adj} + Texture_{adj})/2}{\mathrm{Max}(Face_{adj}, Object_{adj}, Texture_{adj})}$$

Where

$$Face_{adj} = F_{pc} + \left|minimum(F_{pc}, O_{pc,} T_{pc})\right| ;$$

$$Object_{adj} = O_{pc} + \left|minimum(F_{pc}, O_{pc,} T_{pc})\right|;$$

$$Texture_{adj} = T_{pc} + \left|minimum(F_{pc}, O_{pc,} T_{pc})\right|;$$

and F_{pc}, O_{pc} , T_{pc} are percent signal change for faces, objects, or textures, respectively, relative to baseline. This formula differs from FSI_A in that the both positive and negative percent signal change values are adjusted by adding the minimum, whereas FSI_A only adjusts the negative percent change values (the other difference, of course, is in scaling to the maximum rather than the range). The distribution of FSI_B and OSI_B in children and adults is shown in Figures A1 and A2. Again, the FSI_B distribution is negatively skewed in adults and the OSI_B distribution is negatively skewed in children.

Figures A1 and A2 also show the distribution of the Face – Texture and Object – Texture percent signal change as these values could also be used instead of FSI and OSI to test changes in face and object specialization. Similar to the FSI_{JGB} and OSI_{JGB} measures, the distributions are fairly kurtotic due to some extreme values. Although all distributions for FSI (or F – T) are skewed, we further consider the less kurtotic distributions.

FSI_A and FSI_B are virtually identical (Pearson r=.991, p=.0001, n=969 in adults; r=.995, p=.0001, n=760 in children); however, the cases where they deviate illustrate marked differences in the two formulas.

An illustrative case is shown in Figure A3. Percent signal change for all three conditions is shown for a single subject in one brain region (a) and in another brain region (b). Inspection of

the graphs indicates that face specialization should be greater in region (b) than in region (a). Although faces yield a greater signal than either objects or textures in region (a), the difference is somewhat modest compared to the profile in (b) where the face signal is even greater than the average of the object and texture signal. However, FSI_A indicates the same degree of specialization in both cases (~.54). In contrast, FSI_B indicates greater specialization (.541) in region (b) than in region (a)(.382). Because of its apparently greater face validity, FSI_B and OSI_B were used in the present study as the primary measure (referred to in the main text as FSI and OSI), but analyses were also conducted with FSI_A , FSI_{JGB} and the F-O percent signal change (as well as the OSI counterparts). Detailed results are presented in Appendix B.

Figure Captions.

Figure A1. The distributions of different calculation of FSI and OSI, for adults. The distribution of FSI_A, $FSI_{B,}$ FSI_{JGB} and face versus texture percent signal changes were more positively skewed than OSI in adults. This is expected because the ROIs were defined as face-preferential at the voxel level so the FSIs would naturally indicate a greater preference for faces.

Figure A2. The distributions of different calculation of FSI and OSI, for children. The distribution of FSI_A, $FSI_{B,}$ FSI_{JGB} and face versus texture percent signal changes were less positively skewed than in adults, but children have more extreme values (mostly positive values) and the distributions are less kurtotic than in adults.

Figure A3. Percent signal change for all three conditions is shown for a single subject in one brain region (a) and in another brain region (b). Although faces yield a greater signal than either objects or textures in region (a), the difference is somewhat modest compared to the profile in (b).

However, FSI_A indicates the same degree of specialization in both cases (~.54). In contrast, FSI_B indicates greater face specialization (0.541) in region (b) than in region (a)(0.382).

References

Collins, H.R., Zhu, X., Bhatt, R.S., Clark, J.D., Joseph, J.E., 2012. Process and domain specificity in regions engaged for face processing: an fMRI study of perceptual differentiation. J Cogn Neurosci 24, 2428-2444.

Gathers, A.D., Bhatt, R., Corbly, C.R., Farley, A.B., Joseph, J.E., 2004. Developmental shifts in cortical loci for face and object recognition. Neuroreport 15, 1549-1553.

Golarai, G., Ghahremani, D.G., Whitfield-Gabrieli, S., Reiss, A., Eberhardt, J.L., Gabrieli, J.D., Grill-Spector, K., 2007. Differential development of high-level visual cortex correlates with category-specific ecognition memory. Nat Neurosci 10, 512-522.

Joseph, J.E., Cerullo, M.A., Farley, A.B., Steinmetz, N.A., Mier, C.R., 2006. fMRI correlates of cortical specialization and generalization for letter processing. Neuroimage 32, 806-820.

Joseph, J.E., Gathers, A.D., 2002. Natural and manufactured objects activate the fusiform face area. Neuroreport 13, 935-938.

Joseph, J.E., Gathers, A.D., 2003. Effects of structural similarity on neural substrates for object recognition. Cogn Affect Behav Neurosci 3, 1-16.

Joseph, J.E., Gathers, A.D., Bhatt, R.S., 2011. Progressive and regressive developmental changes in neural substrates for face processing: testing specific predictions of the Interactive Specialization account. Dev Sci 14, 227-241.

Simmons, W.K., Bellgowan, P.S., Martin, A., 2007. Measuring selectivity in fMRI data. Nat Neurosci 10, 4-5.

Fig A1

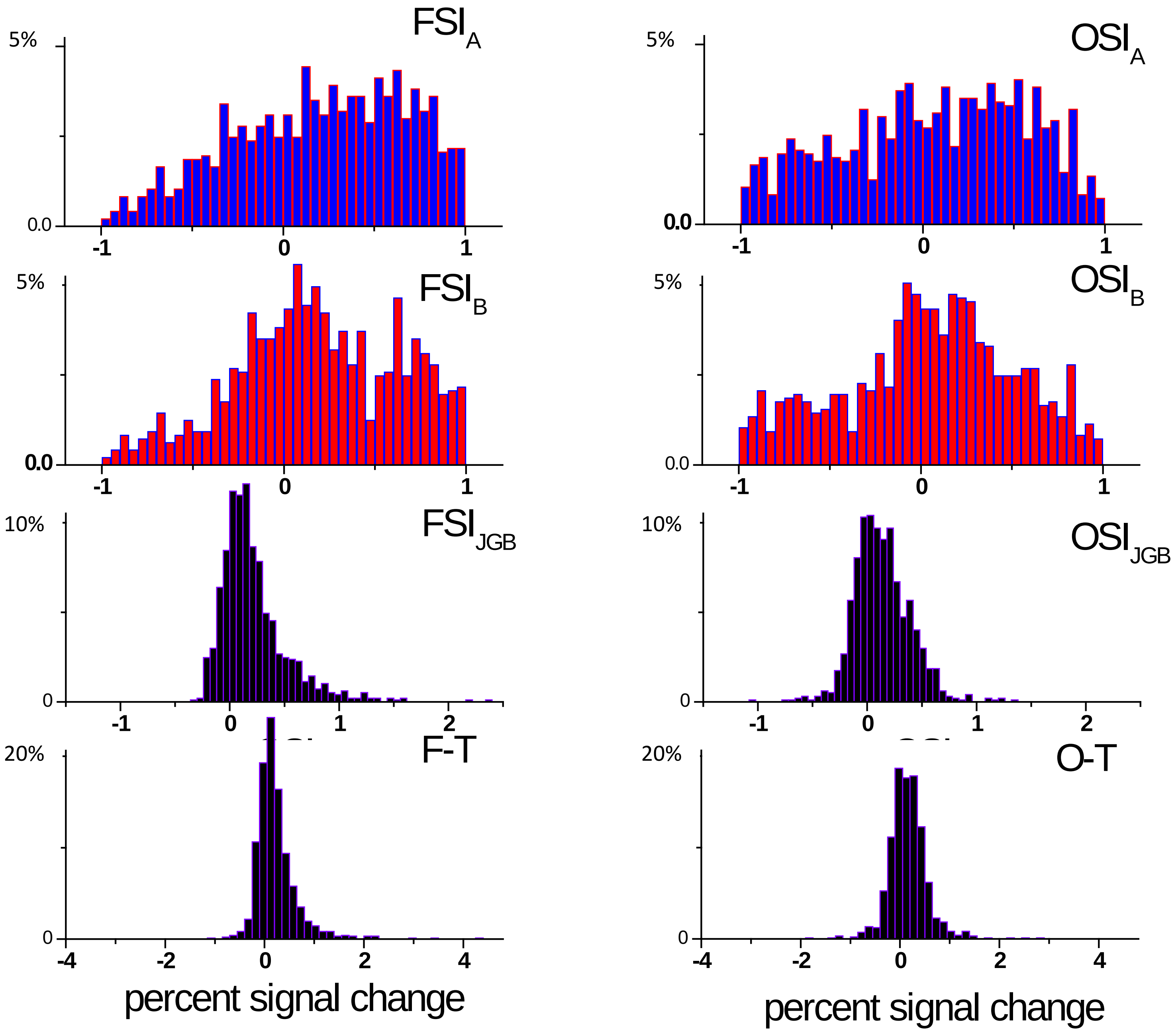
FSIA
5%
0.0
-1
0
1
OSIA
5%
0.0
-1
0
1
FSIB
5%
0.0
-1
0
1
OSIB
5%
0.0
-1
0
1
FSIJGB
10%
0
-1
0
1
2
OSIJGB
10%
0
-1
0
1
2
F-T
20%
0
-4
-2
0
2
4
percent signal change
O-T
20%
0
-4
-2
0
2
4
percent signal change

Fig A2

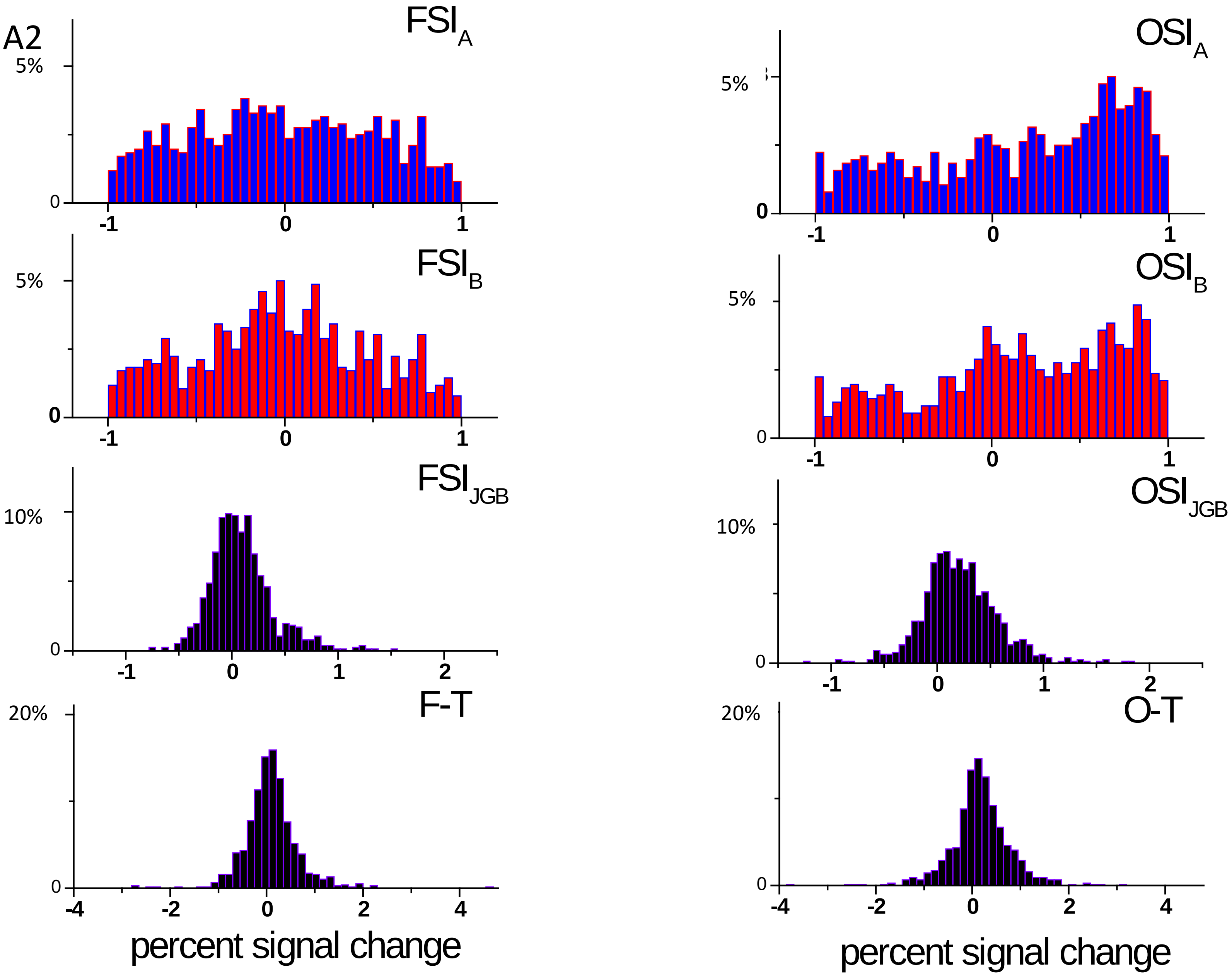

FSI_A
5%
0
-1
0
1
FSI_B
5%
0
-1
0
1
FSI_JGB
10%
0
-1
0
1
2
F-T
20%
0
-4
-2
0
2
4
percent signal change
OSI_A
5%
0
-1
0
1
OSI_B
5%
0
-1
0
1
OSI_JGB
10%
0
-1
0
1
2
O-T
20%
0
-4
-2
0
2
4
percent signal change

Fig A3

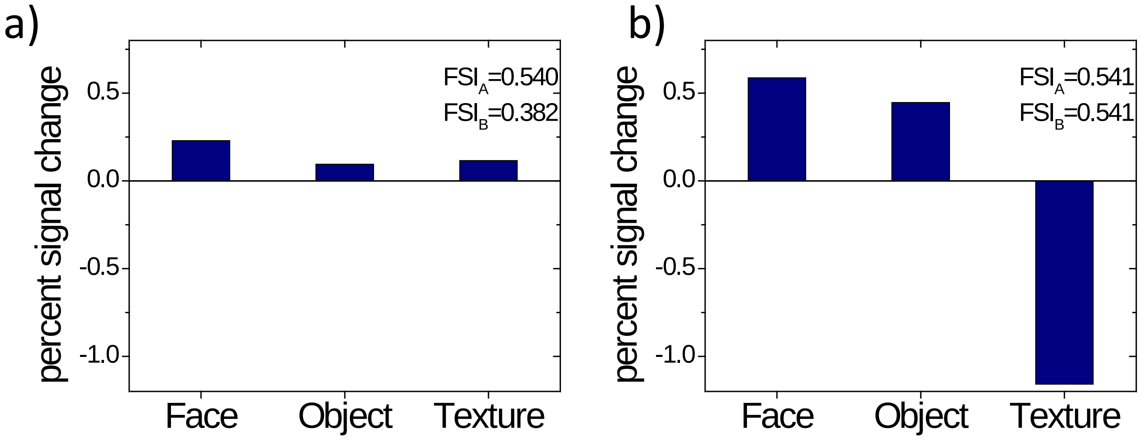
a)
b)
percent signal change
0.5
0.0
-0.5
-1.0
FSI_A=0.540
FSI_B=0.382
FSI_A=0.541
FSI_B=0.541
Face
Object
Texture

Appendix B

Results from the repeated measures ANOVAs conducted using different specialization indices

Following the primary analysis, for each different measure of face specialization, in each ROI, a 2 (category: FSI, OSI) x 3 (age: younger children, older children, adults) repeated measures ANOVA was conducted. In order to compare the sensitivity of the different measures to each main effect or the interaction, Tables B1 through B3 show the results for the main effect of category, main effect of age and the Category x Age interaction, respectively.

With respect to the main effect of category (Table B1), the different measures yield fairly consistent results, with FSI_B and FSI_A having nearly identical findings. The other two specialization indices detected all of the same category effects detected by FSI_B and FSI_A except for the right FFA. In addition, these two indices detected category effects in the right IFGorb and medPFC whereas FSI_B and FSI_A did not.

None of the indices yielded main effects of age in any ROI.

For the Category x Age interactions (Table B2), FT/OT and FSI_{JGB} yield identical results; with FSI_B and FSI_A detecting interactions in all of the same regions as the other two indices. However, FSI_B and FSI_A detected interactions in more ROIs, such as medPFC and IFGorb.

We note that since ROIs were defined in half of the subjects, but the ANOVA was conducted in all subjects, there is a concern of “double dipping” in which analyses are conducted using the same dataset used to define ROIs (Kriegeskorte et al 2009). The reason for including all adult subjects into the primary ROI analysis was to increase statistical power. However, we

also conducted the same ANOVAs in the half of the adult sample that was not used to define ROIs. As seen in Tables B1 and B2, the results barely change when only half of the sample was used for analysis (right-most column). There were only 2 instances where a main effect or interaction was not detected in half of the adult sample: the main effect of category for IFGoper and the Category x Age interaction in the right amygdala.

References

Kriegeskorte N, Simmons WK, Bellgowan PS, Baker CI. (2009). Circular analysis in systems neuroscience: the dangers of double dipping. Nat Neurosci. 12:535-540.

Figure Caption.

Figure B1. Developmental trajectories in ROIs, based on the half-sample of adults. Compare to Fig. 4 to illustrate similar developmental patterns in the half sample.

Table B1. Main effect of category (p value),

Region	FSI_A	FSI_{JGB}	FT/OT	FSI_B All adult subjects	FSI_B Half of adult sample
	F(1, 88) =	F(1, 88) =	F(1, 88) =	F(1, 88) =	F(1, 63) =
IFGorb	ns	0.010*	0.014*	ns	ns
medPFC	0.067	0.019*	0.047*	0.070	0.057
IFGoper	0.015*	0.030*	0.034*	0.030*	0.069
rAMY	0.001*	0.024	0.014*	0.001*	0.011*
lAMY	ns	ns	ns	ns	ns
rTha	ns	ns	ns	ns	ns
lTha	ns	ns	ns	ns	ns
pSTS	<0.001*	<0.001*	<0.001*	<0.001*	<0.001*
FFA	0.01*	0.076	0.080	0.017*	0.048*
MT	<0.001*	<0.001*	<0.001*	<0.001*	<0.001*
rOFA	ns	ns	ns	ns	ns
lOFA	ns	ns	ns	ns	ns
rOP	<0.001*	<0.001*	<0.001*	<0.001*	<0.001*
lOP	<0.001*	<0.001*	<0.001*	<0.001*	<0.001*

*p < 0.05, ns: not significant

Table B2. Category x Age interaction

Region	FSI_A	FSI_{JGB}	FT/OT	FSI_B All adult subjects	FSI_B Half of adult sample
	$F(1, 88) =$	$F(1, 88) =$	$F(1, 88) =$	$F(1, 88) =$	$F(1, 63) =$
IFGorb	0.021*	ns	ns	0.028*	0.049*
medPFC	0.033*	ns	ns	0.040*	0.038*
IFGoper	0.008*	0.029*	0.035*	0.010*	0.050*
rAMY	0.036*	0.033*	0.043*	0.032*	ns
lAMY	0.003*	0.011*	0.016*	0.002*	0.013*
rTha	0.044*	0.015*	0.019*	0.052	0.007*
lTha	0.054	0.003*	0.004*	0.059	0.041*
pSTS	0.026*	0.030*	0.037*	0.048*	0.10
FFA	0.063	0.057	0.067	0.093	ns
MT	0.037*	0.047*	0.01*	0.047*	0.057
rOFA	0.016*	0.025*	0.025*	0.014*	0.028*
lOFA	ns	ns	ns	ns	ns
rOP	ns	ns	ns	ns	ns
lOP	ns	ns	ns	ns	ns

*$p < 0.05$, ns: not significant

Fig B1

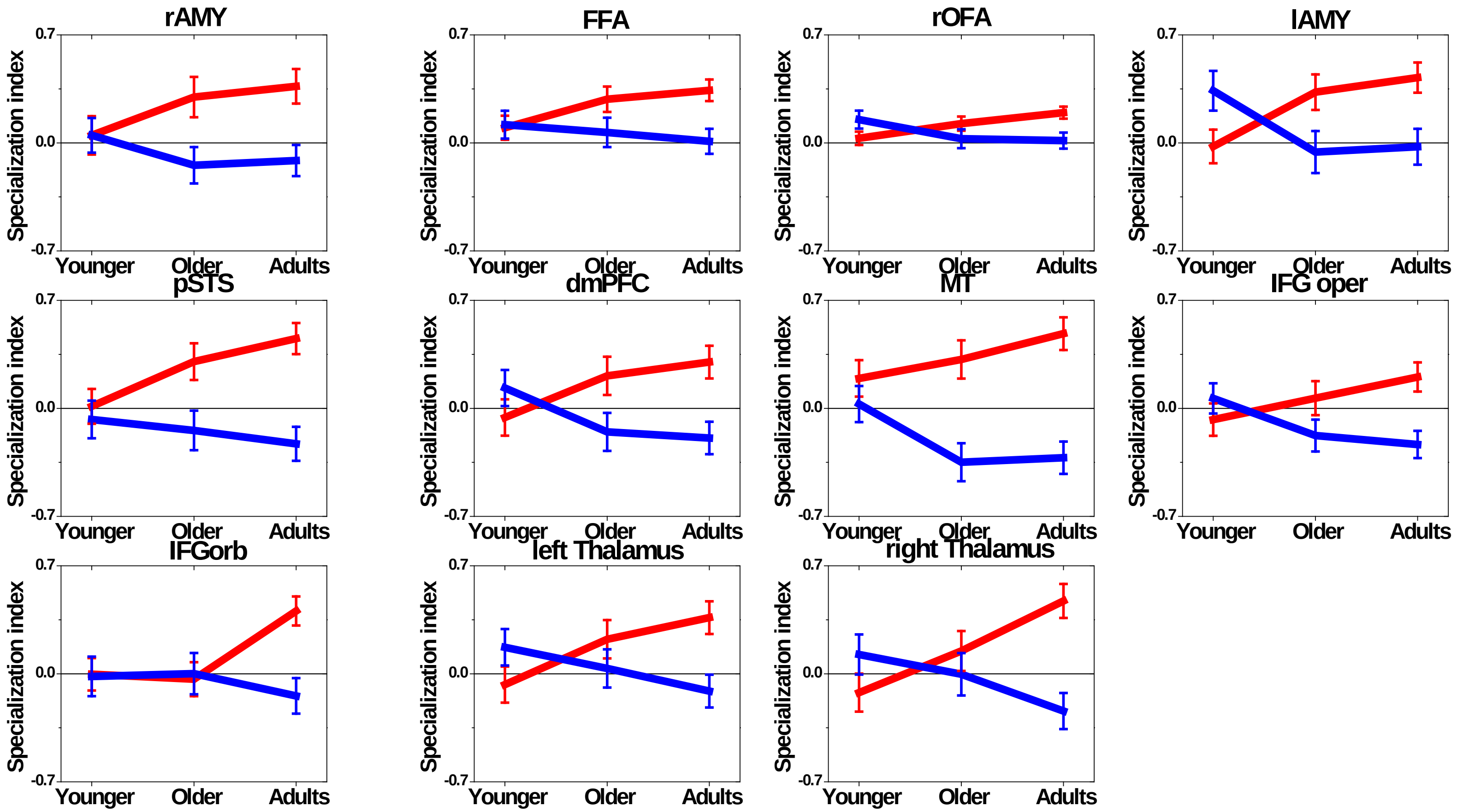
rAMY
FFA
rOFA
lAMY
pSTS
dmPFC
MT
IFG oper
IFGorb
left Thalamus
right Thalamus
Specialization index
0.7
0.0
-0.7
Younger
Older
Adults
